# Tellurite‐Squarate Driven Assembly of a New Family of Nanoscale Clusters Based on (Mo_2_O_2_S_2_)^2+^

**DOI:** 10.1002/chem.201701920

**Published:** 2017-06-29

**Authors:** Jamie W. Purcell, Haralampos N. Miras, De‐Liang Long, Panagiota Markopoulou, Leroy Cronin

**Affiliations:** ^1^ WestCHEM School of Chemistry University of Glasgow Glasgow G12 8QQ UK

**Keywords:** cluster compounds, polyoxothiometalate, self-assembly, tellurite

## Abstract

The preparation and characterization of a new family of four polyoxothiometalate (POTM) clusters are reported, with varying size and complexity, based upon the dimeric [Mo_2_O_2_S_2_(H_2_O)_6_]^2+^ cation with the general formula (NMe_4_)_*a*_K_*b*_[(Mo_2_O_2_S_2_)_*c*_(TeO_4_)_*d*_(C_4_O_4_)_*e*_(OH)_*f*_] where *a*,*b*,*c*,*d*,*e*,*f*={1,7,14,2,4,10}=**1**, {Mo_28_Te_2_}; {2,26,36,12,10,48}=**2**, {Mo_72_Te_12_}; {0,11,15,3,3,21}=**3**, {Mo_30_Te_3_}; {2,6,12,2,4,16}=**4**, {Mo_24_Te_2_}. The incorporation of tellurite anions allowed the fine tuning of the templating and bridging of the available building blocks, leading to new topologies of increased complexity. The structural diversity of this family of compounds ranges from the highly symmetrical cross‐shaped {Mo_24_Te_2_} to the stacked ring structure of {Mo_72_Te_12_}, which is the largest tellurium‐containing POTM cluster reported so far. Also a detailed experimental analysis revealed that the pH isolation window extends from acidic to basic values. ESI‐MS analyses not only confirmed the stability of this family in solution but also revealed the stability of the observed virtual building blocks.

## Introduction

Polyoxometalates (POMs) are molecular metal‐oxide clusters that attract the interest of research groups due to their nano‐scale size, unique structures and wide range of chemical properties.^1^ The fact that almost any element in the periodic table can be incorporated into a POM‐based framework^2^ renders them as exceptional candidates with highly modular structures and functionality. On the other hand, polyoxothiometalates (POTMs) is an under‐studied subset of POM chemistry which emerged by the incorporation of chalcogens;^3^ the combination of a wide range of applications available to metal chalcogenides such as electronics,^4^ hydrogen evolution^5^ and batteries,^6^ with the structural diversity of POMs renders POTMs a unique family of compounds, which offers the opportunity for further exploration and discoveries. The incorporation of chalcogen elements into POTMs, for example, oxygen bridges are replaced by sulfur, alters their behaviour and chemistry which leads to structures and properties not observed in conventional POMs.^7^ The most common precursor utilised for the construction of large clusters is the dinuclear cation [Mo_2_O_2_S_2_]^2+^,^8^ due to its reactivity and flexibility to co‐ordinate to appropriate templates, and generate libraries of building blocks.^9^ Earlier work by Cadot et al. demonstrated that the dimer co‐ordinates easily to carboxylates and that organic molecules with multiple carboxyl groups can be used to expand the ring‐shaped molecules that the dimer usually forms.^10^ Work carried out in our group revealed that certain templates can increase the nuclearity and complexity much further than had been previously observed, with squaric acid, C_4_O_4_H_2_, or selenite, SeO_3_^2−^, both granting access to new generations of building block libraries by acting either as templates or linkers; leading not only to the formation of new and unanticipated structures, but also hugely increasing the size and structural diversity of these compounds.^11^, ^12^

In order to fully utilise the potential of these compounds, it is necessary to understand their formation mechanism. The assembly of POM‐based clusters proceeds through the acid condensation of the parent mononuclear metal‐oxide anion, which can be influenced by a number of variables such as concentration and pH.^13^ This process has also been shown to occur in stages; rather than the full molecules forming directly from the mononuclear starting materials, their molecular growth proceeds through the formation of smaller building units. These species, some of which cannot be isolated,^14^ manifest themselves as structural motifs that repeatedly occur in many different POM structures and are referred to as the building blocks (BBs) that make up the structure.^15^ This concept was very eloquently illustrated by Müller et al. when describing the structures of the partially reduced “Molybdenum Blue” and Keplerate structures.^16^ Self‐assembly processes enable the entire field of supramolecular chemistry, where it has been well established that pre‐organised building blocks can spontaneously assemble into much larger and more complex architectures that straddle the line between discrete molecules and bulk materials.^17^, ^18^ As such, identifying and determining the properties of viable building blocks^19^ is a key aspect of this kind of chemistry and forms a critical part of the work reported here.

As such, we report a family of new nanosized clusters, namely; {Mo_28_Te_2_}=(NMe_4_)K_7_[(Mo_2_O_2_S_2_)_14_(TeO_4_)_2_(C_4_O_4_)_4_(OH)_20_] (**1**), {Mo_72_Te_12_}=(NMe_4_)_2_K_26_[(Mo_2_O_2_S_2_)_36_(Te_3_O_10_)_4_(C_4_O_4_)_10_(OH)_48_] (**2**), {Mo_30_Te_3_}=K_11_[(Mo_2_O_2_S_2_)_15_(TeO_4_)_3_(C_4_O_4_)_3_O(OH)_21_] (**3**) and {Mo_24_Te_2_}=(NMe_4_)_2_K_6_[(Mo_2_O_2_S_2_)_12_(TeO_4_)_2_(C_4_O_4_)_4_(OH)_16_] (**4**).

## Results and Discussion

**Figure 1:**
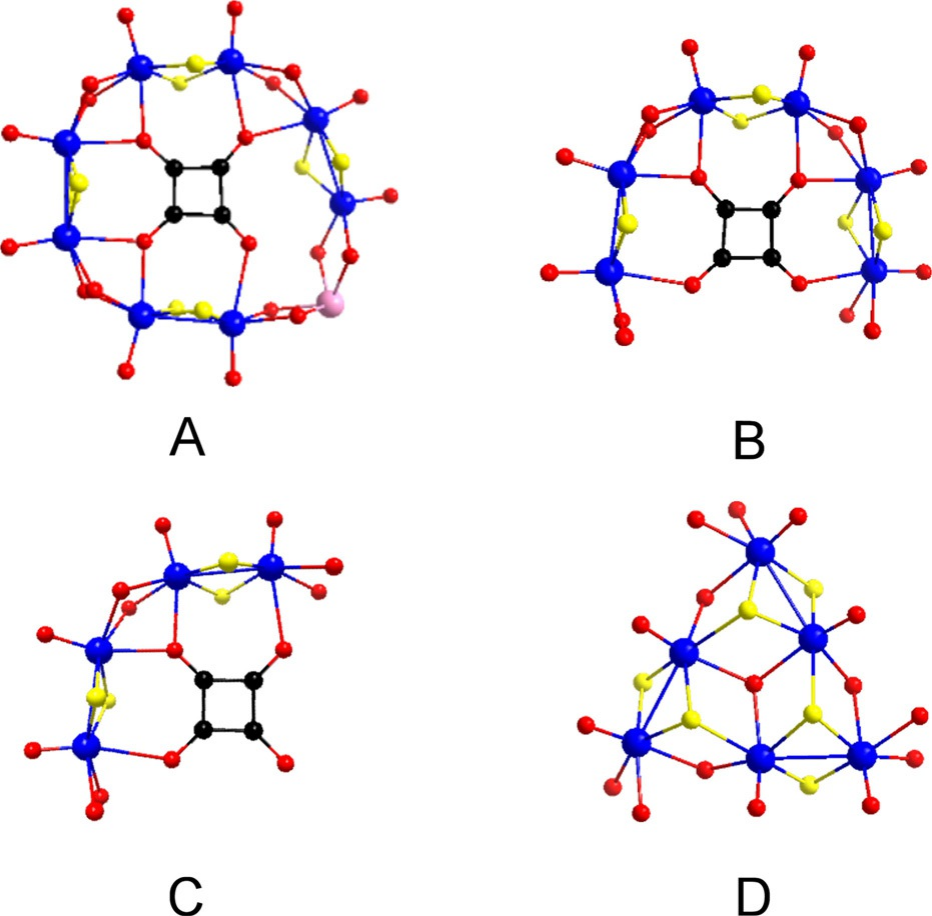
The members of the building block library that have been utilised for the formation of new clusters are: [(Mo_2_O_2_S_2_)_4_(C_4_O_4_)(TeO_3_)(OH)_6_]^2−^, (**A**); [(Mo_2_O_2_S_2_)_3_(C_4_O_4_)(OH)_8_]^4−^, (**B**); [(Mo_2_O_2_S_2_)_2_(C_4_O_4_)(OH)_6_]^4−^, (**C**); [(Mo_2_O_2_S_2_)_3_(O)(OH)_9_]^5−^, (**D**); colour scheme: Mo‐blue, S‐yellow, O‐red, C‐black, Te‐pink.

All of the molecules that are reported herein are derived from a common set of precursors, such as [Mo_2_O_2_S_2_]^2+^ dimer units, C_4_O_4_^2−^ and TeO_3_^2−^ anions (Figure 1). Even though the tellurite and squarate anions can act theoretically as templates, as well as linkers, and generate building block libraries that have been observed before, there are no structural correlations to the final products. Thus, the presence and identity of the tellurite anion is revealed to be a critical factor in the formation of the final structure. In this case the tellurite anions act as linkers between the squarate‐templated building blocks.

The BB **A** is unique to the tellurite/squarate system (Figure 1), which comprises four [Mo_2_O_2_S_2_]^2+^ dimer units connected through pairs of co‐ordinated hydroxide (OH^−^) groups, with Mo−O bond lengths of around 2.1 Å, and a single squarate ion in the centre, co‐ordinated to seven of the eight present Mo atoms through all four O atoms of the squarate, with the average Mo−O bond distance falling at approximately 2.3 Å. The complete ring is formed by a tellurite anion, with the Mo−O bond length here being slightly shorter at 2.0 Å. In contrast, building block **B** is one [Mo_2_O_2_S_2_]^2+^ dimer unit shorter, while the squarate anion is co‐ordinated to Mo centres through all four O atoms. BB **C** can be considered a derivative of building block **A,** which contains two [Mo_2_O_2_S_2_]^2+^dimer units. This leaves one of the four squarate oxygen atoms free and uncoordinated. An interesting variation in bond length has been observed between the three squarate O atoms that are co‐ordinated to Mo; the two outer atoms have notably longer interactions than the central one, 2.5 and 2.2 Å respectively. **D** is a very rare building block that has only been observed in two previous instances to our knowledge.12a, 20 It is a hexameric building block consisting of three dimer units, arranged in a triangular formation, centred on a single μ_3_‐O atom (Mo−O bond length approx. 2.2 Å). In addition to three μ‐OH links, the dimer units are also connected through Mo‐μ_3_S bonds of around 2.6 Å (standard Mo−S bond lengths in the dimer are typically, 2.3–2.4 Å).

**Figure 2:**
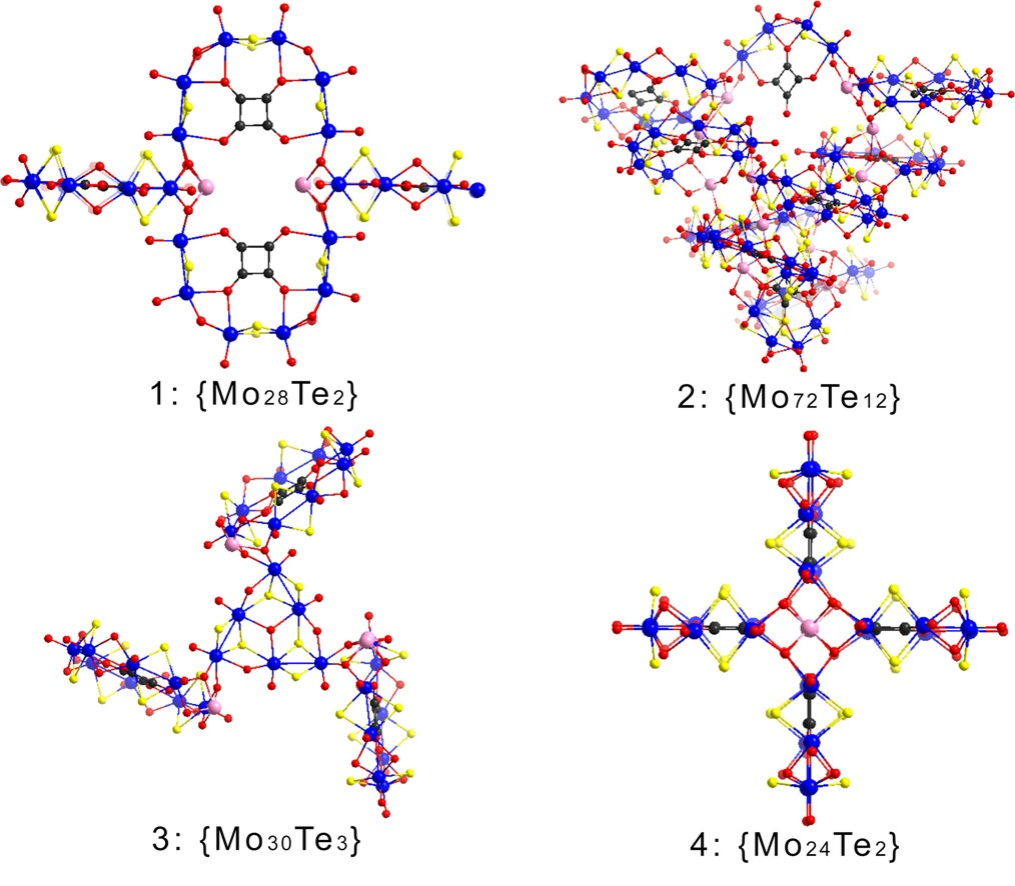
Structural representations of the four newly discovered Te‐containing POTMs: {Mo_28_Te_2_}, **1**; {Mo_72_Te_12_}, **2**; {Mo_30_Te_3_}, **3** and {Mo_24_Te_2_}, **4**; colour scheme: Mo‐blue, S‐yellow, O‐red, C‐black, Te‐pink.

**Figure 3:**
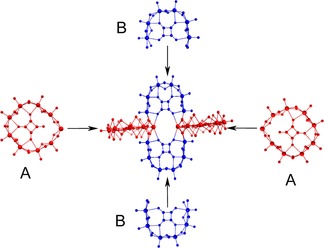
Ball‐and‐stick representation of the formation of **1** through orthogonal assembly of two **A** and two **B** BBs.

These building blocks have been observed to form a number of different structures that have been successfully synthesised and characterised (Figure 2). Cluster **1**, {Mo_28_Te_2_}, comprises two **A** units connected further to two **B** units through the tellurite units, oriented perpendicular to the **A** units to form a cross‐like structure with dimensions 15.6×25.5 Å (Figure 3) that crystallises in the monoclinic system space group *C*2/*c*. Cluster **1** has a *D*_2*h*_ point group, indicating three 2‐fold rotation axes passing through the centre of the molecule, one bisecting the **B** building blocks, one bisecting the **A** building blocks and the third through the open cavity. Each of these axes is also contained within a mirror plane.

**Figure 4:**
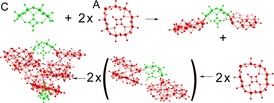
Schematic representation of the sequential build‐up of **2** from the BBs, starting with two **A** connecting with a **C** unit to form the first layer, followed by two additional **A** units to form a second layer, where two of these formations assemble orthogonally to form a four‐layered structure. BB **C** is denoted in green.

**Figure 5:**
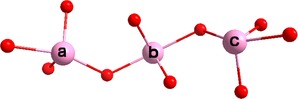
Ball‐and‐stick representation of the {Te_3_O_10_} chain formation present in **2**.

Structure **2**, {Mo_72_Te_12_}, is the largest and most complex of this family. It is constructed by **A** and **C** building blocks; initially a **C** unit bridges two **A** BBs. Two additional **A** units are connected through the tellurite anions, arranged parallel to the first two, creating in effect multiple layers of BBs. An explanation for the size and complex architecture of **2** lies in how the cluster is assembled from the available building blocks. As the building blocks combine, see Figure 4, the intermediates formed at each stage have an accessible side, resulting in very labile species that must continue building in order to form a stable topology. A notable feature of this compound is the Te‐bridge between the building blocks that effectively creates a trapped trimeric Te−O chain formation (Figure 5). It consists of three Te‐atoms connected through O‐bridges, with Te(a) being connected to Te(b) through an O‐bridge forming an angle of 118.5° while the Te(a)−O and Te(b)−O bond lengths were found to be 2.24 and 2.07 Å respectively. In a similar fashion, Te(b) is connected to Te(c) through an O‐bridge forming an angle of 116.2°, with Te(b)−O and Te(c)−O bond lengths of 2.13 and 2.12 Å respectively.

The two half‐structures are joined through the tellurite units of the second set of **A** building blocks, connected through hydroxide linkers to the other half. In the final structure, the two halves are oriented at approximately a 90° angle to each other, with the open ends of each half towards the centre of the molecule, to form a layered‐ring structure of dimensions 24.0×26.6 Å that crystallises in the monoclinic system *C*2/*c* space group (Figure 4). The point group of this molecule is *S*_4_, meaning it has fewer symmetry elements than the previous example; more specifically, it incorporates a 2‐fold rotation axis and a 4‐fold improper rotation axis that bisect the **C** building block in both halves of the structure.

**Figure 6:**
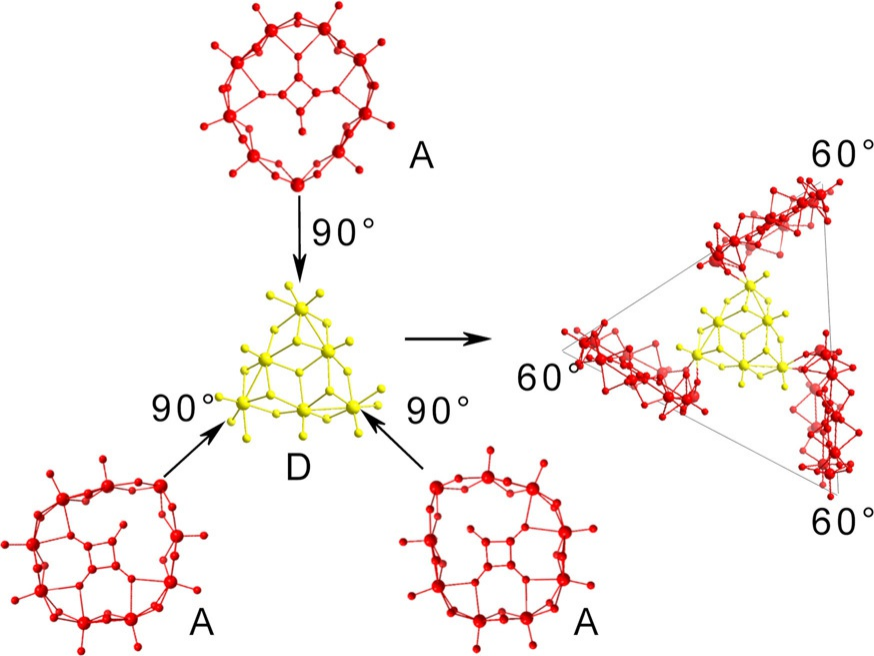
Illustration showing how three **A** units combined with **D** to form compound **3** with their relevant orientations. **D** is denoted in yellow.

Compound **3**, {Mo_30_Te_3_}, is a propeller‐shaped molecule with 3‐fold symmetry, centred on one equivalent of **D** units, with the “blades” of the propeller being three **A** building blocks co‐ordinating through the tellurite group of **A** to each vertex of **D**′s triangular structure, with the length of the sides of the triangular topology being 20.5 Å (Figure 6). **3** displays *C*_3_ symmetry, meaning that the only symmetry element present is a 3‐fold rotation axis through the μ_3_‐O atom of the **D** building block since the space occupied by the three **A** building blocks is not distributed equally on both sides of the central **D** unit, which would be a pre‐requisite for reflection to be a symmetry operation for this molecule. Like the previous two examples, this compound also crystallises in the monoclinic system space group *C*2/*c*.

Compound **4**, {Mo_24_Te_2_}, is a highly symmetrical and aesthetically pleasing cross‐shaped molecule of 15.4 Å in diameter, with several similarities to **1**. The main difference between the two is that **1** is constructed by two different BBs in contrast to **4** which is composed of one; four **B**‐type building blocks are arranged in a similar fashion to that observed in **1**. This change in BBs results in a slightly smaller nuclearity in **4** than in **1** (24 and 28 Mo centres, respectively).

**Figure 7:**
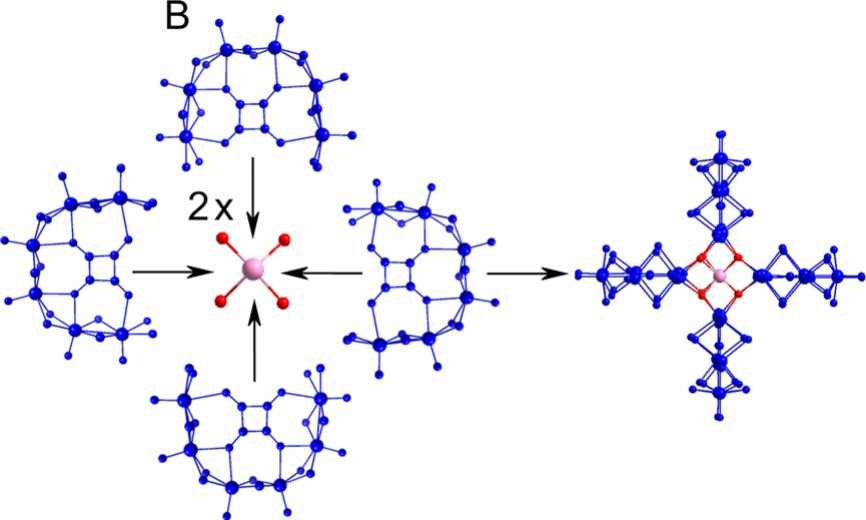
Tellurite templated assembly of four **B**‐type building blocks to form compound **4**.

As seen in **2**, tellurite becomes a building block in its own right in this molecule in order to link the other building blocks together (Figure 7). This compound has the point group *D*_4*h*_, making it the most highly symmetrical structure of the four, which is further reinforced by this compound crystallising in the tetragonal system space group *I*4*cm*. The molecular symmetry elements include a 4‐fold rotation axis passing through both Te‐atoms, four 2‐fold rotation axes (two bisecting the two opposing pairs of building blocks, the other two going between them) and the relevant mirror planes associated with these axes.

A point of interest when comparing **1** and **4** is the difference in orientation of the building blocks, where two of the BBs have been rotated by 90°. It is possible that the size and shape of the cavity in the centre of both of these molecules is important in revealing why the building blocks are arranged in this manner. The central cavity in both of these molecules is identical in both size and shape, with the distance between the two Te‐atoms being 5.3 Å while the shortest distance between carbon atoms on opposing squarate ions is 6.6 Å in both cases. We speculate that it may be able to take up smaller ionic species; however more work would be required to establish whether this is the case.

**Figure 8:**
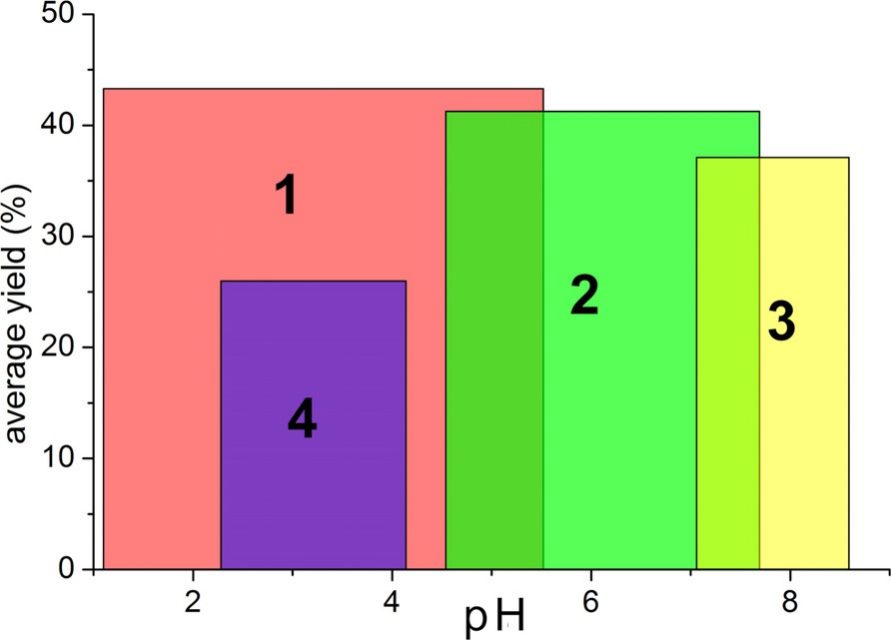
Representation of pH versus average yield for each compound. Red indicates compound **1**, green indicates compound **2**, yellow indicates compound **3**, purple indicates compound **4**.

One of the most interesting aspects of these molecules is the process by which they form. For each structure, the reaction is carried out at room temperature and all reagents are added in the same order. The same volume of water is added to each reaction as solvent and each reaction is carried out for the same length of time. The factors that have been observed to have the greatest bearing on the outcome of the reaction are the ratio of the starting materials and the pH, which is unsurprising considering that it is well established that the condensation reaction that causes POMs to form is triggered by changes in pH. During the course of this work, many reactions were performed over a large pH range in an effort to investigate the whole parameter space. Thus, it is possible to map the areas that are most favourable for each compound. Figure 8 shows the pH ranges where each compound has been observed, along with the average yield obtained. Compounds **1** and **2** occur across a greater range of pH values than the others, with **1** being ubiquitous at lower pH values (1.0–5.5) and **2** appearing to be more common at higher pH values (4.5–7.7) than **1**. It is noteworthy that **2** is the only compound in the set capable of forming at both acidic and basic pH values. The other compounds are more restricted by the pH, with **3** and **4** both forming with a pH window of less than two units. **4** assembles between pH 2.2–4.1. Surprisingly, compound **3** can be formed exclusively at pH values >7, appearing between pH 7.0 and 8.8.

**Figure 9:**
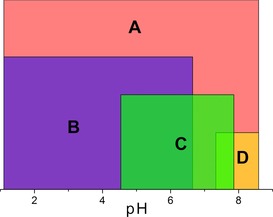
Illustration of where on the pH scale the various BBs that make up the compounds have been observed. Red indicates **A**, purple indicates **B**, green indicates **C**, yellow indicates **D**.

A more fundamental understanding of how this system works could be gained from looking at the pH ranges that the individual BBs occur in, as shown in Figure 9. These pH regions resulted by combining the pH ranges identified for each of the compounds constructed by a specific set of building blocks, for example, BB **A** appears in compounds **1**, **2** and **3**, so the pH window for **A** type building blocks is the total range in which these compounds are found. **A** type building blocks, which appear in three of the four reported compounds, has, unsurprisingly, the broadest range and it occurs across the entire range of pH values investigated. **B** type building blocks extend from the lowest investigated pH across almost the entire range of acidic pH values. **C** has a narrower range than **B**, and is shifted higher up the pH scale. Finally, **D** has the narrowest range of the four BBs and only occurs in one compound at the higher end of the pH range in question.

The IR spectra (Figures S1–4 in Supporting Information) of all of these compounds are very similar since all are formed from the same set of building blocks with identical modes of bonding and interaction. In all four spectra, there is a broad signal at 3400 cm^−1^, indicative of the stretching of the O−H bonds in water, arising from the solvent molecules in the crystal structure and atmosphere. Also arising from the solvent are the signals that appear at 1600 cm^−1^, which correspond to bending vibrations of the water molecules. Signals at 2350 cm^−1^ are assigned to atmospheric CO_2_. The signals at 1520 cm^−1^ are assigned to the C−O bonds of the squarate template. Other signals of interest occur between 480 and 500 (Mo‐S‐Mo bridges), 690 (Mo‐O‐Mo bridges) and 950 cm^−1^ (Mo=O bonds).

Thermogravimetric analysis (TGA, Figures S5–8) experiments show that all four compounds lose around 10–15 % of their mass by 150 °C. This mass drop is assigned to crystallographic water content. Three of the four compounds experience a mass drop between 200 and 400–450 °C, accounting for 10–11 % of the total mass of the sample; in compound **2**, this is manifested over two overlapping steps, accounting for around 14 % of the total mass of the sample. For all of these compounds, the weight loss is associated with the removal of carbon and sulfur content in the form of CO_2_ and SO_2_, respectively.

**Figure 10:**
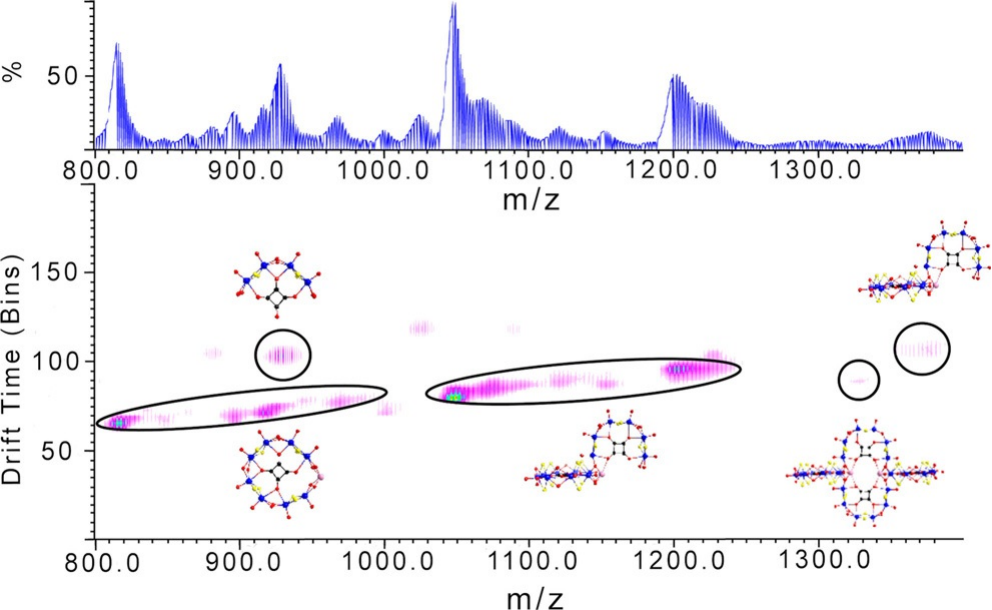
Top: ESI mass spectrum of **1**; bottom: ion mobility ESI mass spectrum of **1** with peak assignments.

Mass spectrometry experiments^21^ showed that not only are these compounds stable in solution, through our observation of the intact clusters, but also we were able to identify the virtual BBs during the fragmentation process (Figure 10). The distribution envelopes that have been tentatively assigned (Figures S9–12 and tables S1–4 in the Supporting Information) have almost universally been either one of the BBs or a collection of multiple BBs that form a fragment of the distinct clusters. The clarity with which the building blocks appear in the mass spectrometry experiments suggests a sequential mechanism of assembly, with the BBs forming first and subsequently combining into the structures that crystallise.

## Conclusion

In conclusion, we have investigated the interaction of [Mo_2_O_2_S_2_]^2+^ units with the tellurite anion for the first time and directed their assembly into a new family of nanoscale structures. Tellurite has been shown to act as a linker species, rather than a true template, when the system also includes the squarate anion. Four new clusters have been successfully synthesised and characterised that represent the structural diversity inherent to POM chemistry. Interesting structural features include the adoption of high levels of symmetry, accessible cavities that could potentially be used to uptake small ions and degrees of complexity that could not have been anticipated. Carefull investigation of the system allowed us to map the reaction co‐ordinates and identify the formation areas of specific building blocks. While there are overlaping areas, it was possible to identify clear pH ranges that favour the formation of each compound, and infer from this a picture of conditions that are preferred by the building blocks that form the basis of each of these structures.

## Experimental Section

All reagents were purchased from Sigma Aldrich, Fisher Scientific and Alfa Aesar, and were used as provided with no further purification required. The (Mo_2_O_2_S_2_)^2+^ dimeric unit was prepared according to the modified procedure published by Cadot et al. in 1998.7a

**X‐ray crystallography**: Data were collected at 150(2) K using a Bruker AXS Apex II (λ(Mo_Kα_)=0.71073 Å) equipped with a graphite monochromator. Structures were solved and refined using Direct methods with SHELXS‐2014^22^ and SHELXL‐2014^23^ using WinGX routines.^24^ Refinement was achieved by full‐matrix least squares on *F* ^2^ through SHELXL. Corrections for incident and diffracted beam absorption effects were applied using analytical methods.^25^ All data manipulation and presentation steps were performed using WinGX. Details of interest about the structure refinement are given in the tables in the Supporting Information.

**Fourier transform infra‐red spectroscopy**: Samples were prepared as KBr discs and FTIR spectra were collected in transmission mode using a Shimadzu IR Affinity‐1S Fourier Transform Infra‐Red Spectrophotometer. Wavenumbers (ν) are given in cm^−1^.

**Electrospray ionisation mass spectrometry (ESI‐MS)** was performed on a Waters Synapt‐G2 HDMS spectrometer operating in ion mobility mode, equipped with a quadrupole and time of flight (Q/ToF) module for MS analysis. All samples were prepared by dissolving in 1:10 H_2_O:MeCN (HPLC grade) to a concentration of ca. 1×10^−5^ m and injected directly at a flow rate of 5 μL min^−1^ using a Harvard syringe pump. All spectra were collected in negative ion mode and analysed using the Waters MassLynx v4.1 software. For all measurements the following parameters were employed: capillary voltage: 2.5 kV; sample cone voltage: 10.0 V; extraction cone voltage: 4.0 V; source temperature: 80 °C; desolvation temperature: 180 °C; cone gas flow: 15 L h^−1^ (N_2_); desolvation gas flow: 750 L h^−1^ (N_2_).

**Thermogravimetric analysis**: Analysis was performed on a TA Instruments Q500 Thermogravimetric Analyser under nitrogen flow with a typical heating rate of 10 °C min^−1^ from room temperature up to 800 °C.

**Elemental analysis**: Mo, S and Te content were determined by ICP‐OES analysis in the following way: 5–10 mg sample material was digested by adding 1 mL deionised water and 2 mL conc. HNO_3_ to the sample in a digestion beaker. The sample solution was warmed until clear before being allowed to cool and a further 5 mL deionised water added. The resulting solution was transferred quantitatively with washings to an A class 50 mL volumetric flask and made up to the mark with deionised water. A blank sample was also prepared simultaneously to account for any digestion interferences. The samples were transferred to 50 mL polypropylene centrifuge tubes and analysed on an Agilent SVDV 5100 ICP‐OES using the SVDV mode and appropriate calibration standards. Carbon and nitrogen content was analysed by the University of Glasgow microanalysis service within the School of Chemistry. Potassium content was determined using a Corning 410 Flame Photometer using the same samples and calibration standards used in the ICP‐OES analysis.

All TGA and elemental analysis experiments were run on dry samples and as such, some crystallographic water had already been lost, accounting for the discrepancy between the crystallographic water content as stated in the molecular formulae and the water content found in the TGA experiments.

### Synthetic procedures

**(NMe_4_)K_7_[(Mo_2_O_2_S_2_)_14_(TeO_4_)_2_(C_4_O_4_)_4_(OH)_20_]⋅72 H_2_O (1)**: Na_2_TeO_3_ (0.05 g, 0.225 mmol) and C_4_O_4_H_2_ (0.1 g, 0.877 mmol) were dissolved together in 20 mL distilled water to form a cloudy white solution. Dimeric [Mo_2_O_2_S_2_]^2+^ (5 mL, 0.68 mmol) was added to give a black colour to the solution. 1 m K_2_CO_3(aq)_ was used to bring the pH of the solution to 3.3 with the colour turning to clear orange. The reaction mixture was stirred at room temperature for one hour, during which time the pH rose to 4.9. The reaction mixture was then filtered and kept at 18 °C. Within 2 weeks orange block crystals formed that were suitable for crystallography studies. 179.2 mg material was collected (56.88 % yield based on Mo). Elemental analysis calcd (%) for C_20_H_172_K_7_Mo_28_NO_142_S_28_Te_2_ (6812.32): Mo 39.43, S 13.18, Te 3.75, C 3.53, N 0.21, K 4.02; found: Mo 38.73, S 13.24, Te 4.25, C 3.73, N 0.33, K4.47.

**(NMe_4_)_2_K_26_[(Mo_2_O_2_S_2_)_36_(Te_3_O_10_)_4_(C_4_O_4_)_10_(OH)_48_]⋅125 H_2_O (2)**: Na_2_TeO_3_ (0.05 g, 0.225 mmol) and C_4_O_4_H_2_ (0.1 g, 0.877 mmol) were dissolved together in 20 mL distilled water to form a cloudy white solution. Dimeric [Mo_2_O_2_S_2_]^2+^ (6.5 mL, 0.878 mmol) was added to give a black colour to the solution. 1 m K_2_CO_3 (aq)_ was used to bring the pH of the solution to 5.15 with the colour turning to clear orange. The reaction mixture was stirred at room temperature for one hour, during which time the pH rose to 7.17. The reaction mixture was then filtered and kept at 18 °C. Within 2 weeks orange block crystals formed that were suitable for crystallography studies. 240 mg material was collected (54.87 % yield based on Mo). Elemental analysis calcd (%) for C_48_H_172_K_26_Mo_72_N_2_O_250_S_72_Te_12_ (16541.35): Mo 41.76, S 13.96, Te 9.26, C 3.49, N 0.17, K 6.15; found: Mo 41.44, S14.11, Te 9.63, C 2.90, N 0.15, K 6.07.

**K_11_[(Mo_2_O_2_S_2_)_15_(TeO_4_)_3_(C_4_O_4_)_3_O(OH)_21_]⋅70 H_2_O (3)**: Na_2_TeO_3_ (0.05 g, 0.225 mmol) and C_4_O_4_H_2_ (0.1 g, 0.877 mmol) were dissolved together in 20 mL distilled water to form a cloudy white solution. Dimeric [Mo_2_O_2_S_2_]^2+^ (6.5 mL, 0.878 mmol) was added to give a black colour to the solution. 1 m K_2_CO_3 (aq)_ was used to bring the pH of the solution to 5.2 with the colour turning to clear orange. The reaction mixture was stirred at room temperature for one hour, during which time the pH rose to 7.6. The reaction mixture was then filtered and kept at 18 °C. Within 2 weeks red rod‐shaped crystals formed that were suitable for crystallography studies. 230 mg material was collected (58.38 % yield based on Mo). Elemental analysis calcd (%) for C_12_H_141_K_11_Mo_30_O_136_S_30_Te_3_ (7114.97): Mo 40.45, S 13.52, Te 5.38, C 2.03, K 6.04; found: Mo 40.08, S 12.70, Te 4.91, C 2.18, 6.22.

**(NMe_4_)_2_K_6_[(Mo_2_O_2_S_2_)_12_(TeO_4_)_2_(C_4_O_4_)_4_(OH)_16_]⋅55 H_2_O (4)**: Na_2_TeO_3_ (0.05 g, 0.225 mmol) and C_4_O_4_H_2_ (0.1 g, 0.877 mmol) were dissolved together in 20 mL distilled water to form a cloudy white solution. Dimeric [Mo_2_O_2_S_2_]^2+^ (5 mL, 0.68 mmol) was added to give a black colour to the solution. 1 m K_2_CO_3 (aq)_ was used to bring the pH of the solution to 3.5, with the colour turning to clear orange. The reaction mixture was stirred at room temperature for one hour, after which the pH was 2.98. The reaction mixture was then filtered and kept at 18 °C. Within 2 weeks orange rod‐shaped crystals formed that were suitable for crystallography studies. 103.4 mg material was collected (32.41 % yield based on Mo). Elemental analysis calcd (%) for C_24_H_150_K_6_Mo_24_N_2_O_119_S_24_Te_2_ (5933.12): Mo 38.81, S 12.97, Te 4.30, C 4.86, N 0.47, K 3.95; found: Mo 37.72, S 13.32, Te 4.45, C 4.70, N 0.39, K 4.56.

CCDC 1538939 (**1**), 1538940 (**2**), 1538941 (**3**) and 1538942 (**4**) contain the supplementary crystallographic data for this paper. These data are provided free of charge by The Cambridge Crystallographic Data Centre.

**pH studies**: In order to map the pH ranges for each compound, many reactions were performed under a variety of conditions. No significant variation in reaction outcome was observed when the ratios between reagents were changed. All reagents were combined together in the flask, base added and the pH was recorded. After one hour of stirring, the pH of each reaction was recorded once more, which are the values used to establish the optimal pH ranges of each compound. pH was recorded twice due to this value changing over the course of the reaction. A selection of the reaction conditions used, detailing the highest and lowest yield, manually set pH values and final pH values are given in Tables S9–S12 in the Supporting Information. The synthetic procedures given above correspond to the conditions that resulted in the highest yield.

## Conflict of interest

The authors declare no conflict of interest.

## Supporting information

As a service to our authors and readers, this journal provides supporting information supplied by the authors. Such materials are peer reviewed and may be re‐organized for online delivery, but are not copy‐edited or typeset. Technical support issues arising from supporting information (other than missing files) should be addressed to the authors.

Supplementary
