# Supplementary material for: Tellurite‐Squarate Driven Assembly of a New Family of Nanoscale Clusters Based on (Mo2O2S2)2+
Source: Chemistry. 2017 Jun 29;23(40):9683–9. doi: 10.1002/chem.201701920 (PMC5575479; doi:10.1002/chem.201701920)
Supplement: Supplementary file 1 — Supplementary [file CHEM-23-9683-s001.pdf]

CHEMISTRY
A European Journal

Supporting Information

Tellurite-Squarate Driven Assembly of a New Family of Nanoscale Clusters Based on $(Mo_2O_2S_2)^{2+}$

Jamie W. Purcell, Haralampos N. Miras, De-Liang Long, Panagiota Markopoulou, and Leroy Cronin*[a]

chem_201701920_sm_miscellaneous_information.pdf

Supporting Information

Tellurite-Squarate Driven Assembly of a New Family of Nanoscale Clusters based on $(Mo_2O_2S_2)^{2+}$

Jamie W. Purcell, Haralampos N. Miras, De-Liang Long, Panagiota Markopoulou and Leroy Cronin*

S2: FT-IR Data

S4: Thermogravimetric Data

S6: ESI-IM-MS Data

S10: Crystallographic Data

S14: Reaction Conditions

FT-IR

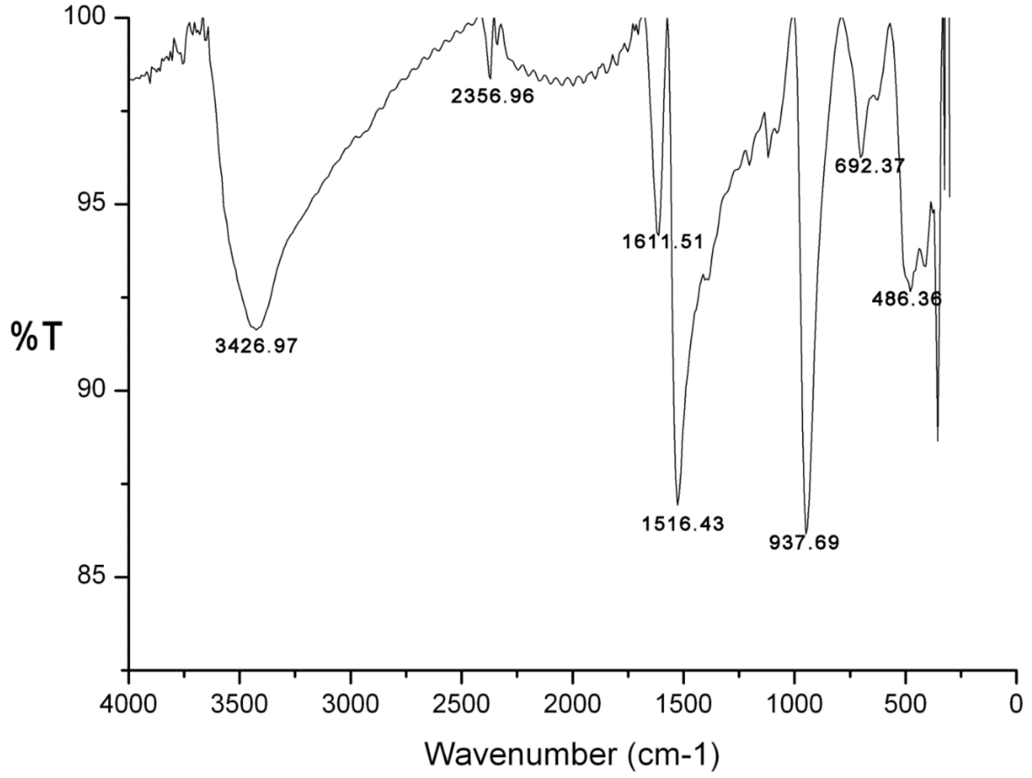

Figure S1: FT-IR Spectrum of **1**

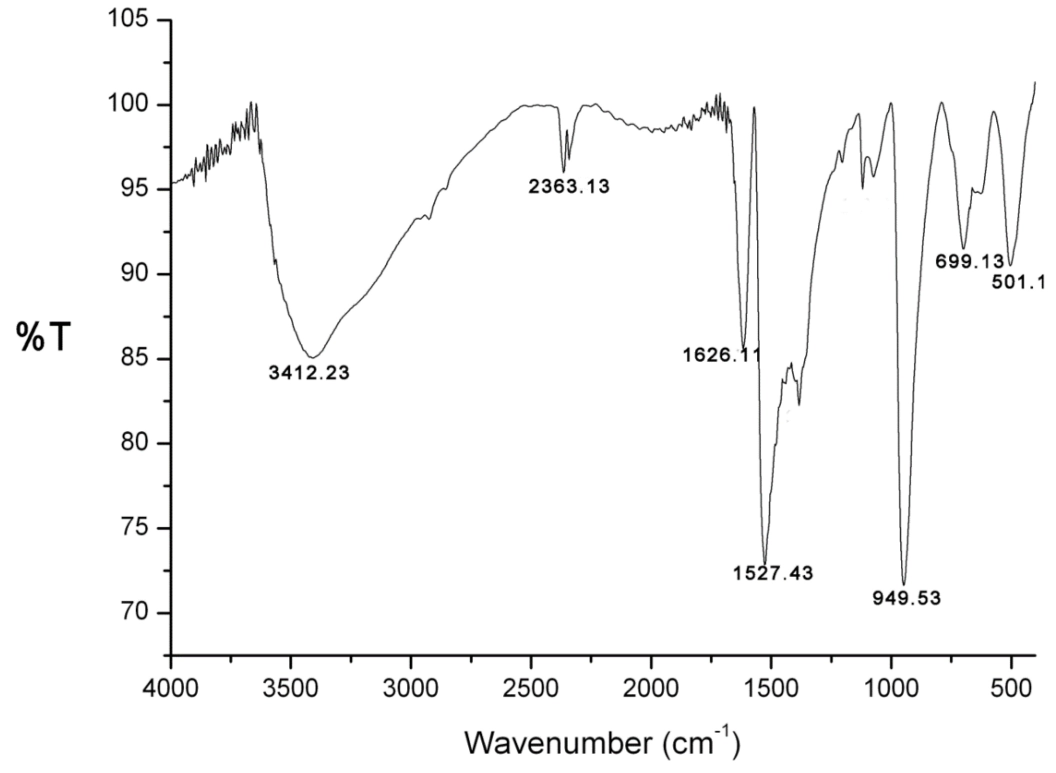

Figure S2: FT-IR spectrum of **2**

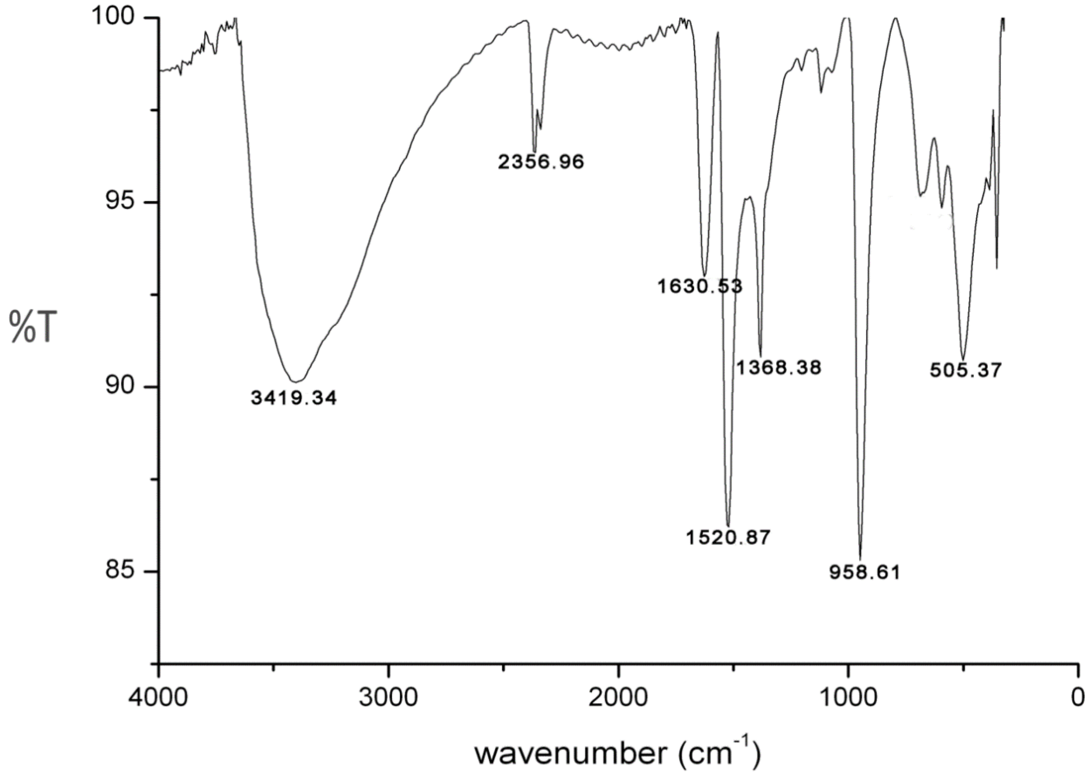

Figure S3: FT-IR Spectrum of **3**

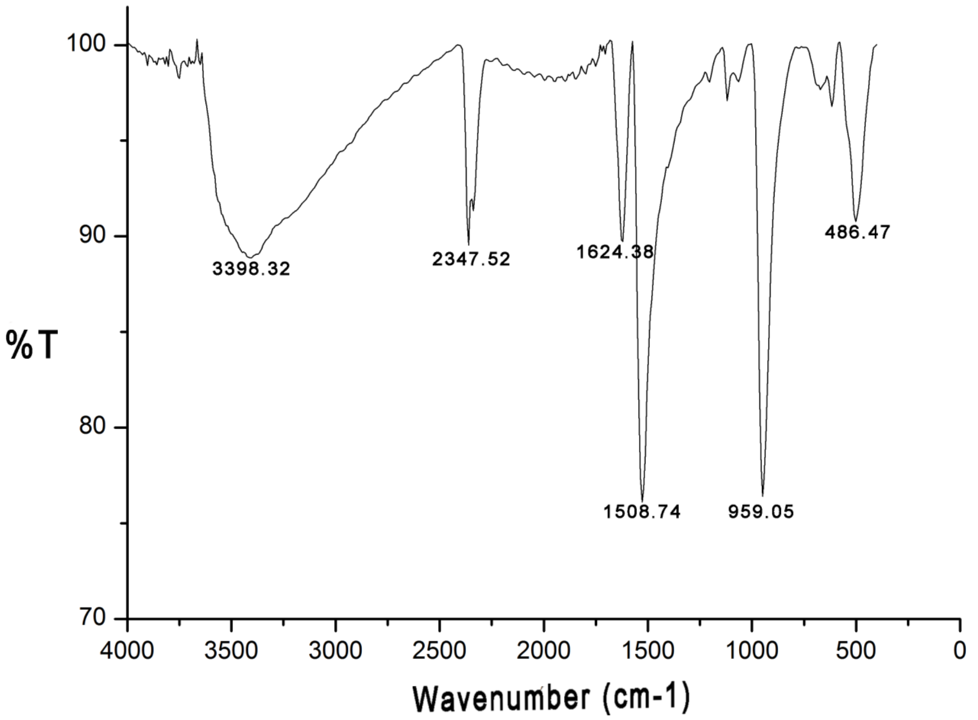

Figure S4: FT-IR Spectrum of **4**

Thermogravimetric Data

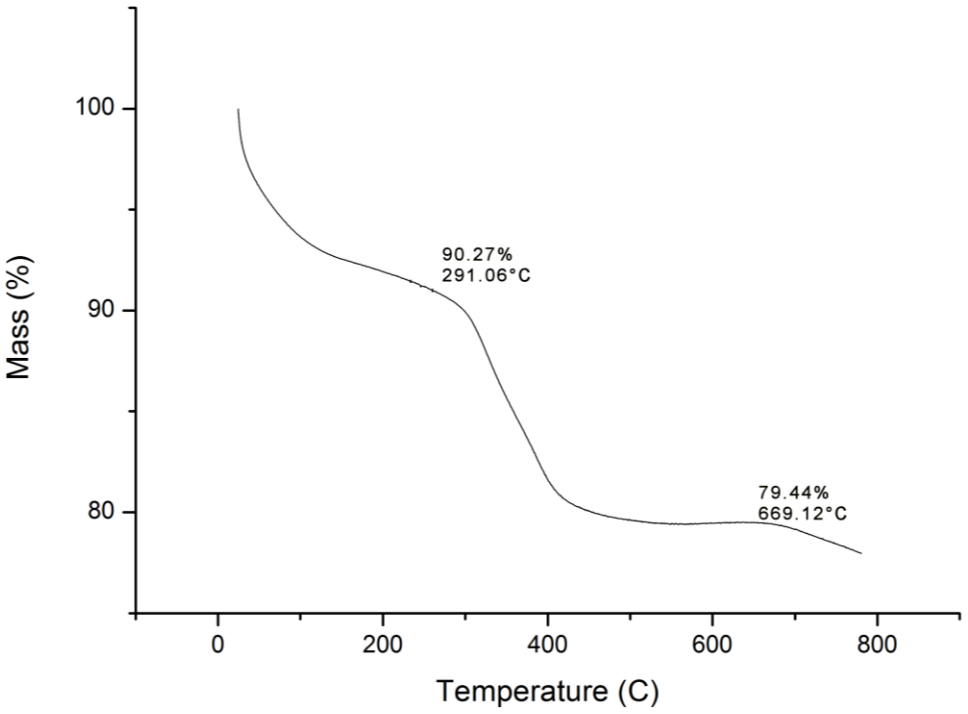

Figure S5: TGA graph of compound **1**

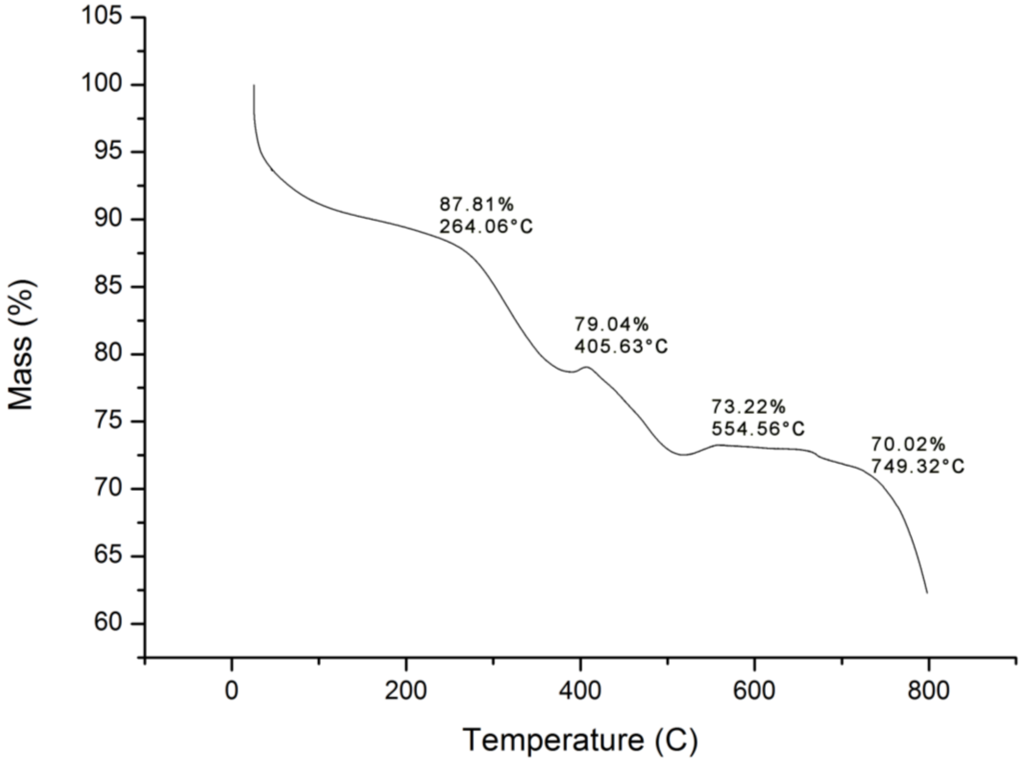

Figure S6: TGA graph of Compound **2**

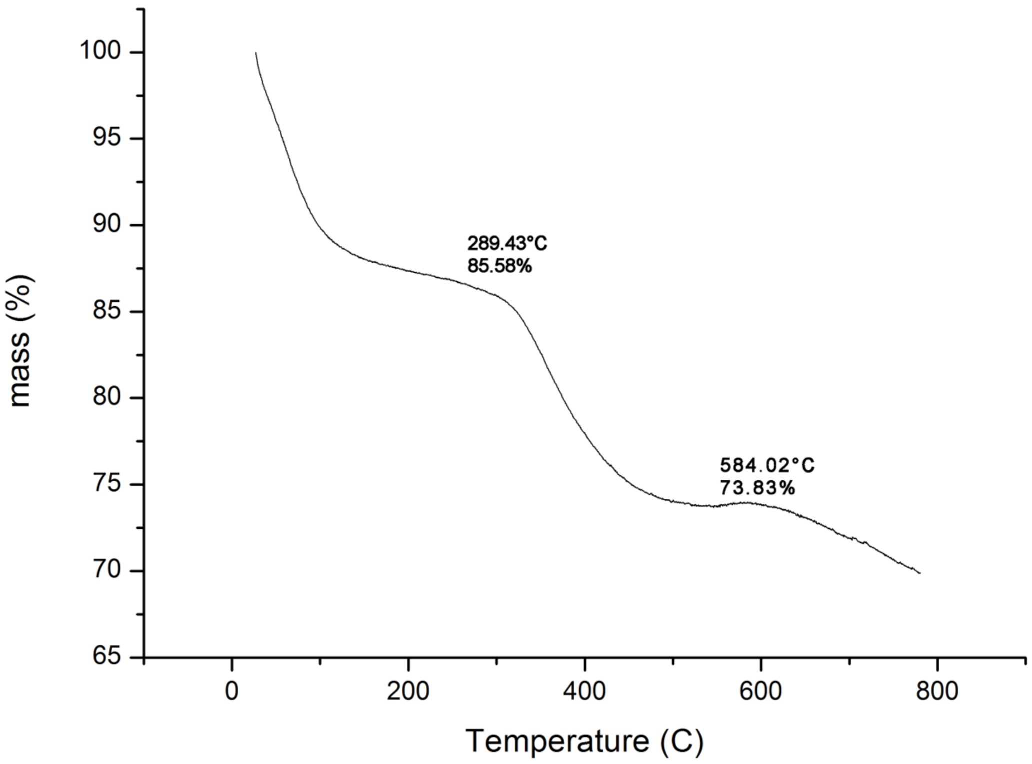

Figure S7: TGA graph of compound **3**

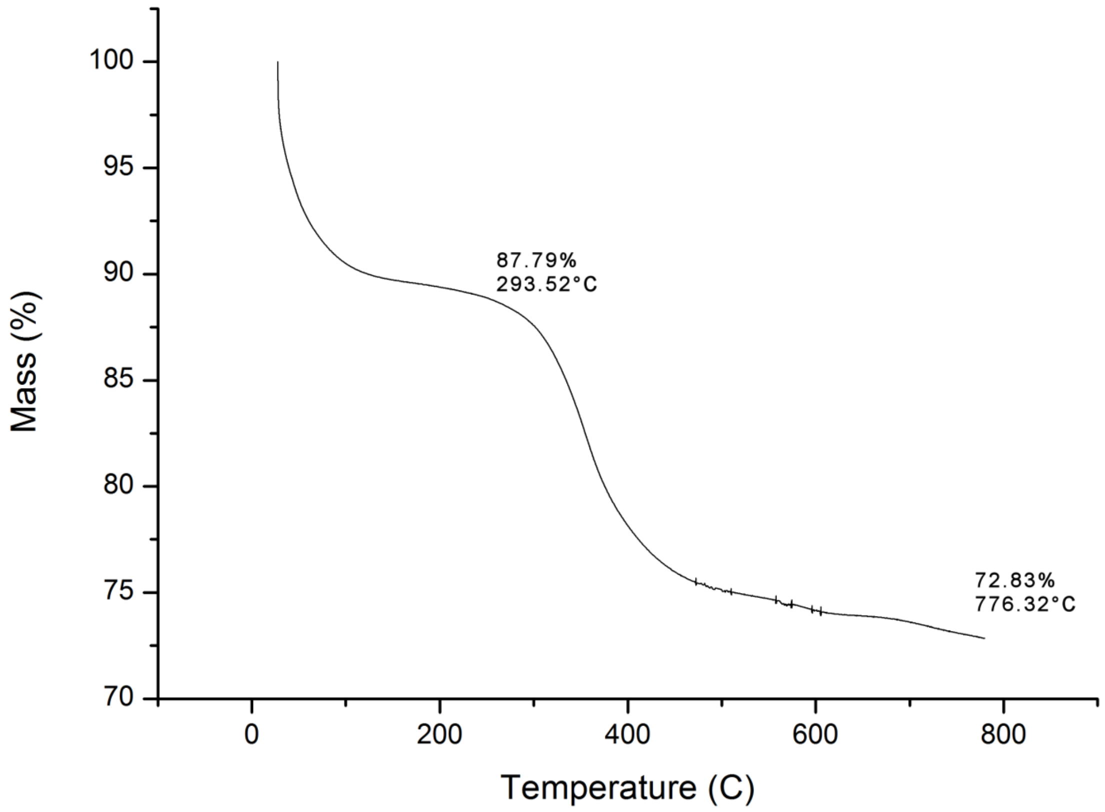

Figure S8: TGA graph of compound **4**

ESI-IM-MS Data

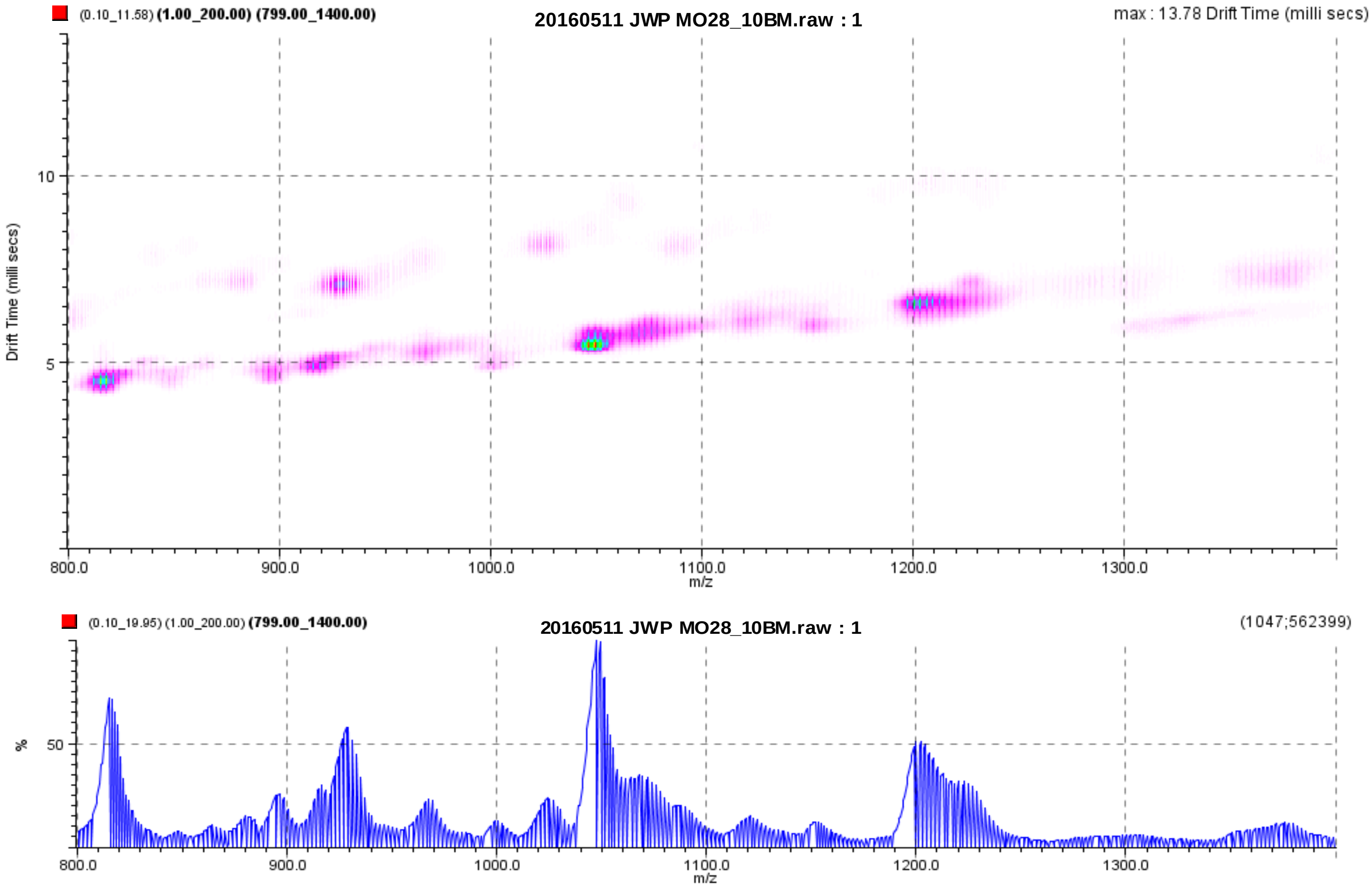

Figure S9: ESI mass spectra of **1**, upper: ESI-IM-MS spectrum, lower: ESI-MS spectrum

Table S1: Tentative Ion Mobility Mass Spectrometry peak assignments for Compound **1**

m/z	z	Tentative Assignment	Comments	Calculated m/z
816.40	-2	$K(Mo_2O_2S_2)_4(C_4O_4)(TeO_3)(OH)_9(H)_2$	BB **A**	816.93
848.35	-2	$Na_3(Mo_2O_2S_2)_4(C_4O_4)(TeO_3)(OH)_9(H_2O)_2$	BB **A**	848.93
896.35	-2	$KNa_4(Mo_2O_2S_2)_4(C_4O_4)(TeO_3)(OH)_{11}(H_2O)_2$	BB **A**	896.91
916.35	-2	$KNa_5(Mo_2O_2S_2)_4(C_4O_4)(TeO_3)(OH)_{12}(H_2O)_2$	BB **A**	916.91
968.78	-2	$K_5Na_2(Mo_2O_2S_2)_4(C_4O_4)(TeO_3)(OH)_{13}(H_2O)_2$	BB **A**	969.35
929.60	-1	$KNa_2(Mo_2O_2S_2)_2(C_4O_4)(OH)_6(H_2O)_3$	BB **C**	930.46
1000.23	-2	$KNa_2(Mo_2O_2S_2)_5(C_4O_4)(TeO_3)(OH)_{11}$	BB **A** + 1 dimer	1000.28
1047.72	-2	$K_4(Mo_2O_2S_2)_5(C_4O_4)_2(OH)_{12}(H_2O)_4$	Half-Molecule- 2 dimer, -1 tellurite	1048.31
1070.23	-2	$(Mo_2O_2S_2)_6(C_4O_4)_2(OH)_{10}(H_2O)$	Half-Molecule- 1 dimer, -1 tellurite	1070.23
1201.11	-2	$K_3Na(Mo_2O_2S_2)_6(C_4O_4)_2(OH)_{14}(H_2O)_4$	Half-Molecule- 1 dimer, -1 tellurite	1201.20
1328.13	-4	$(Mo_2O_2S_2)_{14}(C_4O_4)_4(TeO_3)_2(OH)_{20}(H_2O)_8$	Full Molecule	1329.07
1375.99	-2	$K_3(Mo_2O_2S_2)_7(C_4O_4)_2(TeO_3)(OH)_{13}$	Half-Molecule (1x**A**, 1x**B**)	1377.00

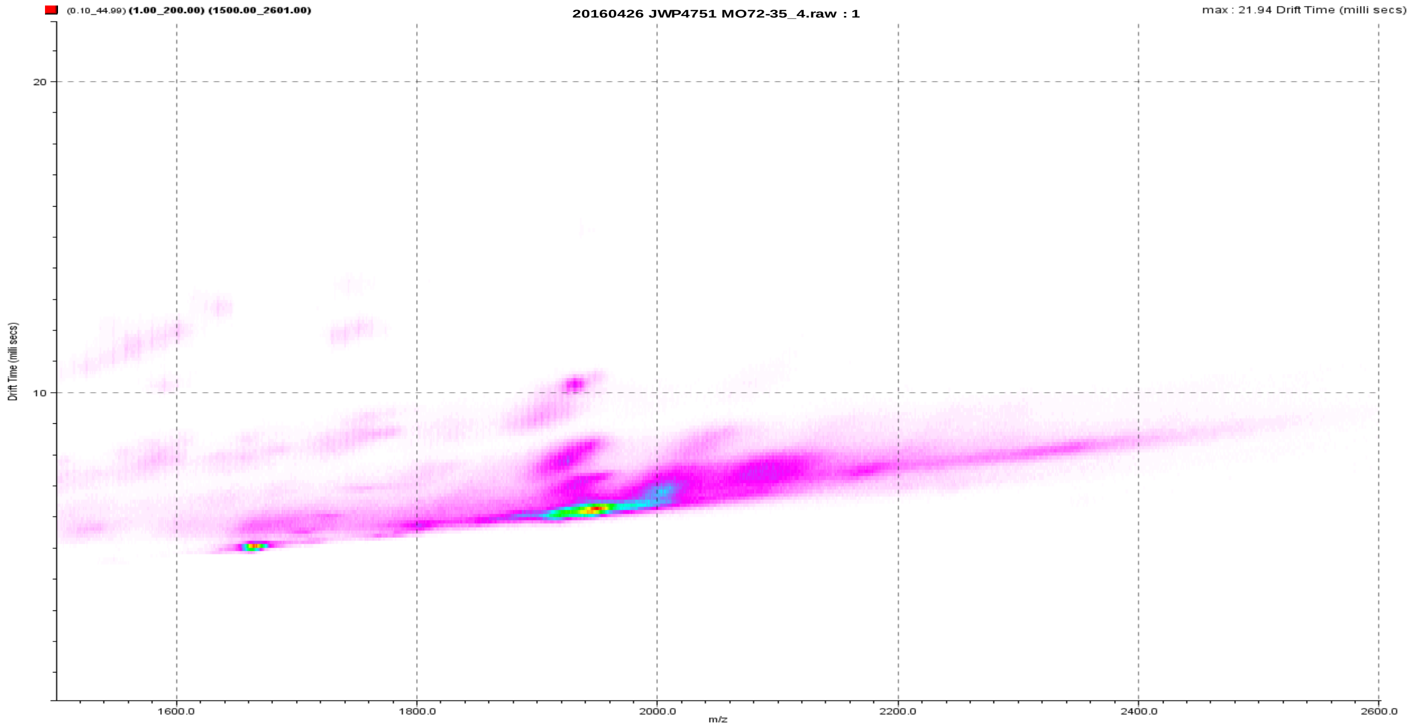

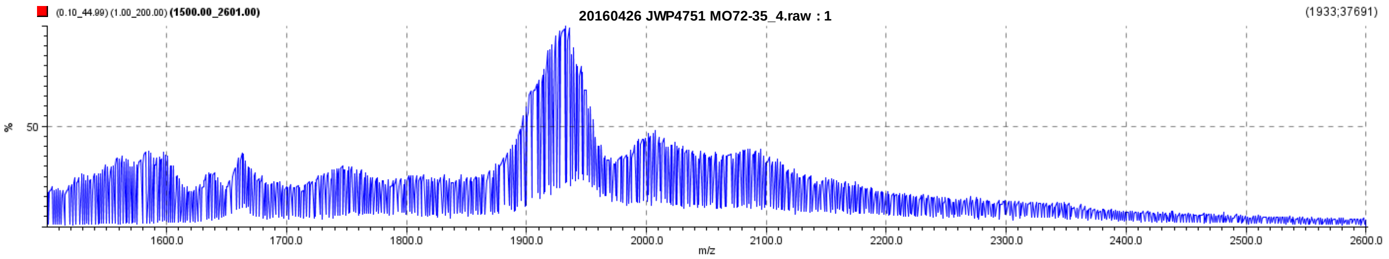

Figure S10: ESI mass spectra of **2**, upper: ESI-IM-MS spectrum, lower: ESI-MS spectrum

Table S2: Tentative Ion Mobility Mass Soectrometry peak assignments for Compound **2**

m/z	z	Tentative Assignment	Comments	Calculated m/z
1665.75	-7	$(Mo_2O_2S_2)_{30}(C_4O_4)_8(Te_3O_{10})_3(OH)_{27}(H_2O)_2$	Full Molecule –BBs **A** + **C**	1665.75
1948.86	-6	$K(Mo_2O_2S_2)_{30}(C_4O_4)_8(Te_3O_{10})_3(OH)_{27}(H_2O)_2$	Full Molecule –BBs **A** + **C**	1949.87
2001.44	-5	$(Mo_2O_2S_2)_{25}(C_4O_4)_7(Te_3O_{10})_3(OH)_{17}(H_2O)_6$	Full Molecule – 2**A**, **C**	2001.92
2089.35	-4	$K_3(Mo_2O_2S_2)_{22}(C_4O_4)_6(Te_3O_{10})(TeO_3)(OH)_{29}(H_2O)$	Full Molecule – 3**A**, **C**	2088.70
1931.03	-4	$K_5(Mo_2O_2S_2)_{20}(C_4O_4)_4(Te_3O_{10})(TeO_3)(OH)_{31}(H_2O)_4$	Full Molecule – 3x**A** + 2x**C**	1930.33
1932.00	-2	$K(Mo_2O_2S_2)_{10}(C_4O_4)_3(TeO_3)_2(OH)_{13}(H_2O)_2$	BB **C** + 2x**A**	1932.09
1926.15	-3	$(Mo_2O_2S_2)_{15}(C_4O_4)_4(Te_3O_{10})(OH)_{17}(H_2O)_{10}$	Half Molecule	1926.64

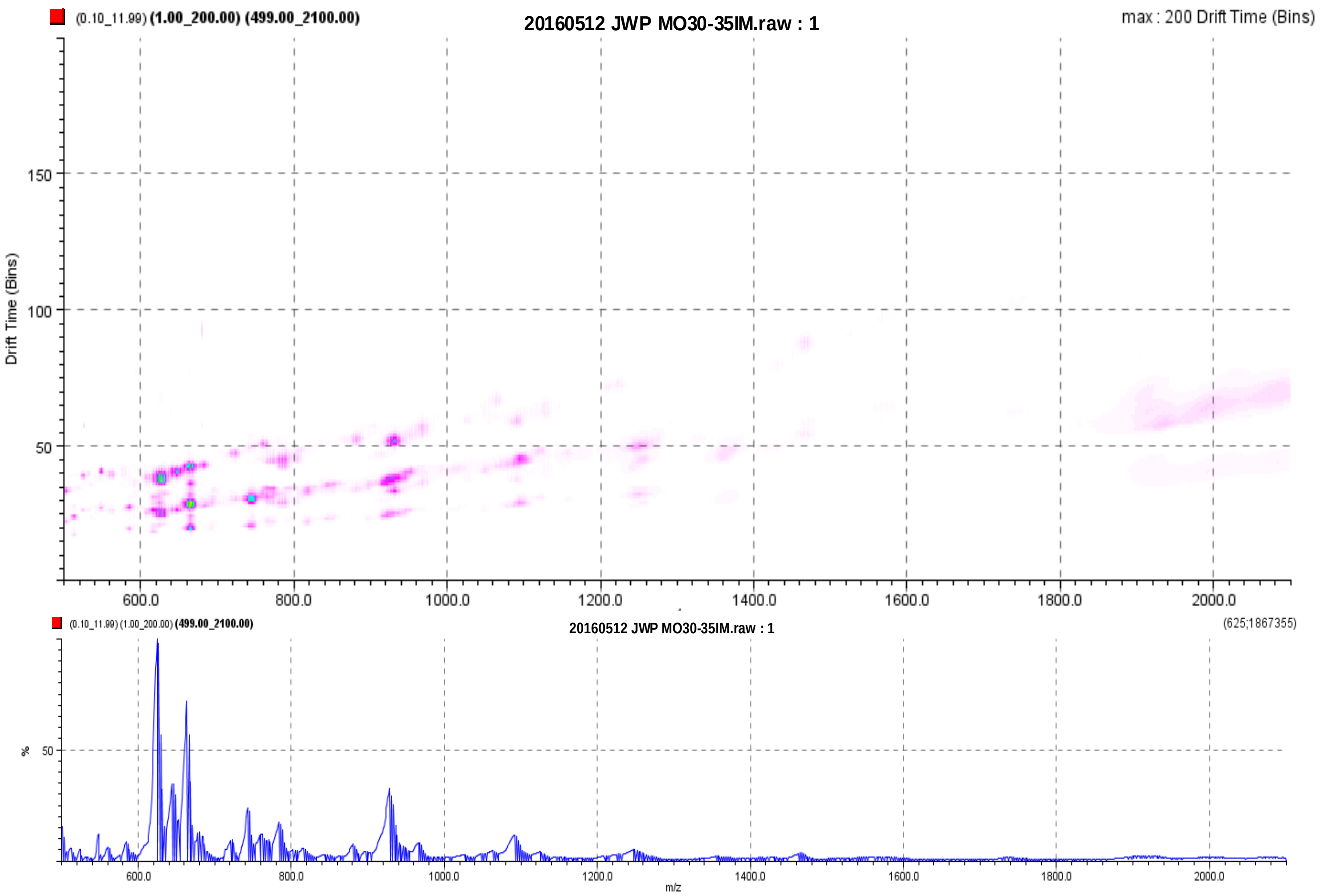

Figure S11: ESI mass spectra of **3**, upper: ESI-IM-MS spectrum, lower: ESI-MS spectrum

Table S3: Tentative Ion Mobility Mass Spectrometry assignments for Compound **3**

m/z	z	Tentative Assignment	Comments	Calculated m/z
624.53	-1	$KNa_2(Mo_2O_2S_2)(C_4O_4)(OH)_4(H_2O)_4$	1 dimer unit + 1 squarate	624.72
626.54	-1	$K(Mo_2O_2S_2)(TeO_3)(OH)_2(H_2O)_5$	1 dimer unit + 1 tellurite	626.66
646.51	-1	$K_2(Mo_2O_2S_2)(TeO_3)(OH)_3(H_2O)_3$	1 dimer unit + 1 tellurite	646.60
663.53	-2	$K_2Na_2(Mo_2O_2S_2)_3(C_4O_4)(OH)_8(H_2O)_5$	BB **B**	663.60
664.50	-1	$K_2(Mo_2O_2S_2)(TeO_3)(OH)_3(H_2O)_4$	1 dimer unit + 1 tellurite	664.61
664.53	-2	$K(Mo_2O_2S_2)_3(C_4O_4)(TeO_3)(OH)_5(H_2O)_3$	BB **A** – 1 dimer	664.56
743.48	-2	$KNa_3(Mo_2O_2S_2)_3(TeO_3)(C_4O_4)(OH)_8(H_2O)_5$	BB **A** – 1 tellurite	743.56
787.43	-1	$K_2Na_2(Mo_2O_2S_2)(TeO_3)(C_4O_4)(OH)_5(H)_2$	1 dimer, 1 squarate, 1 tellurite	786.55
816.40	-2	$K(Mo_2O_2S_2)_4(TeO_3)(C_4O_4)(OH)_7(H_2O)_2$	BB **A**	816.93
845.42	-2	$K_2(Mo_2O_2S_2)_4(TeO_3)(C_4O_4)(OH)_8(H_2O)_2$	BB **A**	845.41
882.43	-1	$KNa_4(Mo_2O_2S_2)(TeO_3)(C_4O_4)(OH)_5(H_2O)_5$	1 dimer, 1 squarate, 1 tellurite	881.60
923.35	-2	$K_4Na_2(Mo_2O_2S_2)_4(TeO_3)(C_4O_4)(OH)_{12}$	BB **A**	923.36
927.35	-2	$Na_2(Mo_2O_2S_2)_5(C_4O_4)(OH)_{13}(H)(H_2O)_2$	BB **A** + 1 dimer - 1 tellurite	928.37
930.30	-1	$Na_2K(Mo_2O_2S_2)_2(C_4O_4)(OH)_6(H_2O)_3$	BB **C**	930.46

1092.20	-1	$K(Mo_2O_2S_2)_3(OH)_7(H_2O)_3(O)$	BB **D**	1093.25
1095.20	-2	$Na(Mo_2O_2S_2)_6(TeO_3)(C_4O_4)(OH)_7(O)_2(H)$	BB **A** + (BB **D** – 1 dimer)	1095.67
1094.71	-2	$Na_4K(Mo_2O_2S_2)_5(TeO_3)(C_4O_4)(OH)_{11}(O)(H_2O)_7$	BB **D** + **C** linked by tellurite	1094.31
1248.6	-2	$K_5(Mo_2O_2S_2)_6(TeO_3)(C_4O_4)(OH)_{14}(O)_3$	BB **A** + (BB **D** – 1 dimer)	1248.59
1249.11	-2	$(Mo_2O_2S_2)_7(C_4O_4)_2(OH)_{12}(H_2O)_3$	BBs **A**+**D**, -1 tellurite	1249.12
1253.14	-3	$(Mo_2O_2S_2)_{10}(TeO_3)_2(C_4O_4)_2(OH)_{17}(H2O)(H)_2$	2x BB **A** linked by (BB **D** – 1 dimer)	1254.76
1360.39	-3	$(Mo_2O_2S_2)_{11}(TeO_3)_2(C_4O_4)_2(OH)_{17}(H_2O)_3$	Full cluster –BB **A**	1362.34

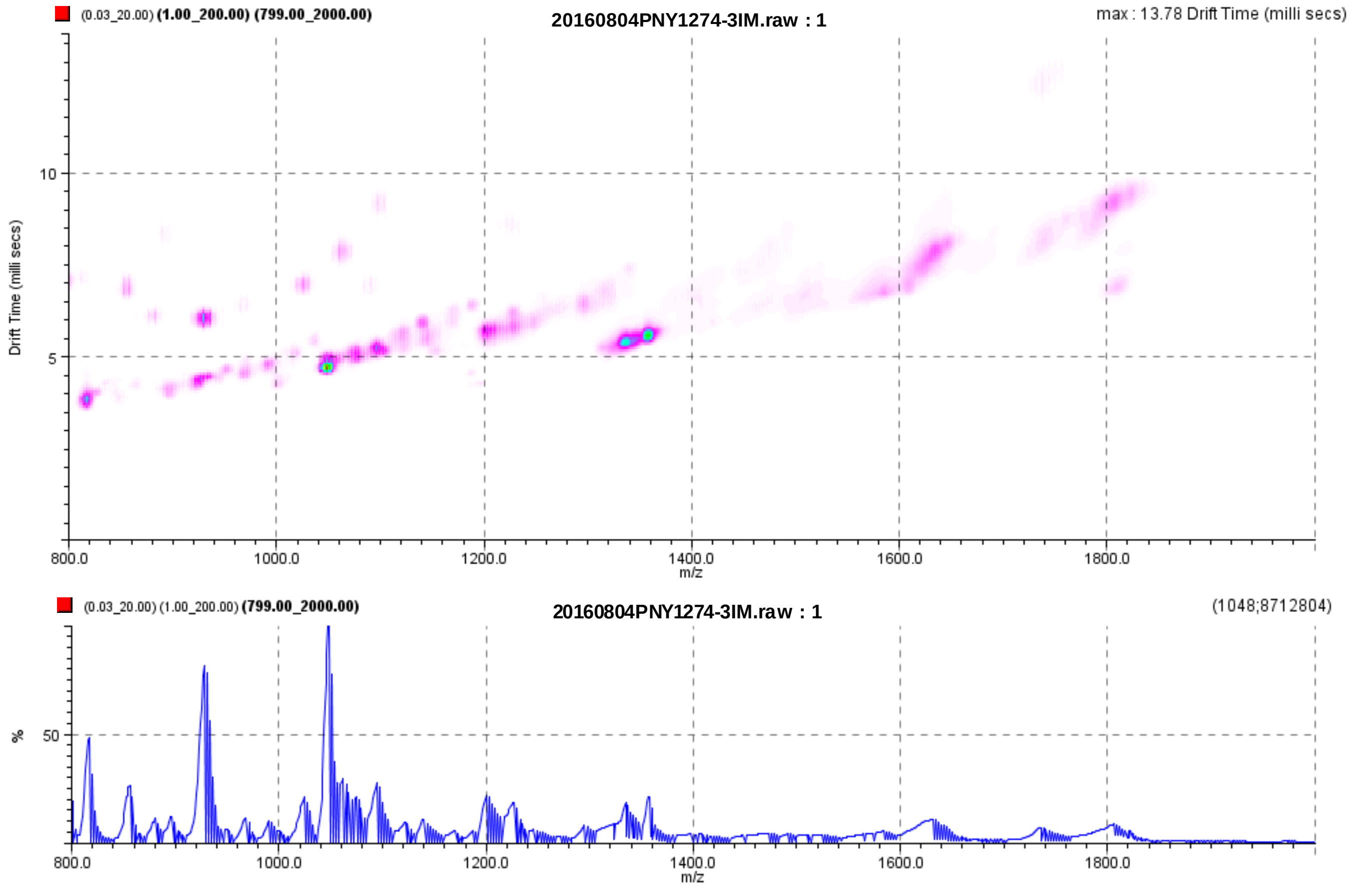

Figure S12: ESI mass spectra of **4**, upper: ESI-IM-MS spectrum, lower: ESI-MS spectrum

Table S4: Tentative Ion Mobility Mass Spectrometry peak assignments for Compound **4**

m/z	z	Tentative Assignment	Comments	Calculated m/z
816.83	-2	$K(Mo_2O_2S_2)_4(TeO_3)(C_4O_4)(OH)_7(H_2O)_2$	BB **A**	816.93
855.40	-1	$KNa(Mo_2O_2S_2)_2(C_4O_4)(OH)_5(H_2O)$	BB **C**	854.45
929.21	-1	$K_2Na(Mo_2O_2S_2)_2(C_4O_4)(OH)_6(H_2O)_2$	BB **C**	928.42
1048.14	-2	$K_4(Mo_2O_2S_2)_5(C_4O_4)_2(OH)_{12}(H_2O)_4$	Half-Molecule, -1 dimer, -1 tellurite	1048.31
1076.15	-2	$K_5(Mo_2O_2S_2)_5(C_4O_4)_2(OH)_{13}(H_2O)_4$	2x BB **B**	1076.29
1095.62	-2	$KNa_3(Mo_2O_2S_2)_5(TeO_3)(C_4O_4)_2(OH)_{10}(H_2O)_4$	Half-Molecule, -1 dimer	1095.29
1063.16	-1	$(Mo_2O_2S_2)_3(C_4O_4)(OH)_5$	BB **B**	1062.23
1201.03	-2	$K5(Mo_2O_2S_2)_5(TeO_3)(C_4O_4)_2(OH)_{11}(H_2O)_{10}$	Half-Molecule	1201.26
1247.01	-2	$K_2Na_2(Mo_2O_2S_2)_6(TeO_3)(C_4O_4)_2(OH)_{12}(H_2O)_2$	Half-Molecule	1246.14
1228.02	-2	$K_2Na_2(Mo_2O_2S_2)_6(TeO_3)(C_4O_4)_2(OH)_{12}$	Half-Molecule	1228.13
1335.03	-4	$K_6Na_2(Mo_2O_2S_2)_{13}(TeO_3)_2(C_4O_4)_4(OH)_{26}(H_2O)_4$	Full Molecule + 1 dimer	1334.56
1357.59	-4	$K_{10}Na(Mo_2O_2S_2)_{13}(TeO_3)_2(C_4O_4)_4(OH)_{28}$	Full Molecule + 1 dimer	1358.52
1584.55	-3	$K_4(Mo_2O_2S_2)_{12}(TeO_3)_2(C_4O_4)_4(OH)_{19}(H_2O)$	Full Molecule	1584.52
1608.88	-3	$K_5Na(Mo_2O_2S_2)_{12}(TeO_3)_2(C_4O_4)_4(OH)_{21}$	Full Molecule	1610.50
1633.52	-3	$K_6(Mo_2O_2S_2)_{12}(TeO_3)_2(C_4O_4)_4(OH)_{21}(H_2O)_3$	Full Molecule	1633.84
1735.06	-3	$K_{12}(Mo_2O_2S_2)_{12}(TeO_3)_2(C_4O_4)_4(OH)_{27}(H_2O)$	Full Molecule	1734.09
1808.10	-3	$K_8(Mo_2O_2S_2)_{13}(TeO_3)_2(C_4O_4)_4(OH)_{26}(H_2O)_7$	Full Molecule + 1 dimer	1808.08
1823.13	-3	$K_{10}Na(Mo_2O_2S_2)_{13}(TeO_3)_2(C_4O_4)_4(OH)_{28}(H_2O)_2$	Full Molecule + 1 dimer	1823.37
1806.09	-3	$K_3Na(Mo_2O_2S_2)_{14}(TeO_3)_2(C_4O_4)_4(OH)_{23}(H_2O)_3$	Full Molecule + 2 dimers	1805.70

Crystallographic Data

Table S5: Crystallographic Data for Compound **1**

Identification code	JWP4292
Empirical formula	C20 H176 K7 Mo28 N O144 S28 Te2
Formula weight	6848.51
Temperature	150(2)K
Wavelength	0.71073Å
Crystal system, space group	Monoclinic, C 2/c
Unit cell dimensions	a = 29.4219(16)Å α = 90°<br>b = 15.3666(9)Å β = 103.254(2)°<br>c = 42.769(2)Å γ = 90°
Volume	18821.3(18)$Å^3$
Z, Calculated density	4, 2.417Mg/m^3
Absorption coefficient	2.669 mm^{-1}
F(000)	13264
Crystal size	0.100 x 0.050 x 0.050 mm
θ range for data collection	1.957 to 26.000°
Limiting indices	-36<=h<=36, -18<=k<=18, -52<=l<=52
Reflections collected / unique	94466 / 18472 [R(int) = 0.0502]
Completeness to θ = 25.242	99.9%
Absorption correction	Empirical
Max. and min. transmission	0.745 and 0.596
Refinement method	Full-matrix least-squares on F^2

Data / restraints / parameters	18472 / 0 / 1021
Goodness-of-fit on F^2	1.037
R indices (all data)	R1 = 0.0426, wR2 = 0.1056
Extinction coefficient	n/a
Largest diff. peak and hole	2.22 and -0.84 e.Å^{-3}

Table S6: Crystallographic Data for Compound **2**

Identification code	JWP4502
Empirical formula	C48 H322 K26 Mo72 N2 O325 S72 Te12
Formula weight	17892.86
Temperature	150(2)K
Wavelength	0.71073Å
Crystal system, space group	Monoclinic, C 2/c
Unit cell dimensions	a = 40.755(3)Å α = 90°<br>b = 40.387(3)Å β = 114.172(3)°<br>c = 33.3692(19)Å γ = 90°
Volume	50108(5) Å^3
Z, Calculated density	4, 2.372 Mg/m^3
Absorption coefficient	3.024 mm^{-1}
F(000)	34072
Crystal size	0.100 x 0.050 x 0.050mm
θ range for data collection	1.320 to 26.000°
Limiting indices	-38<=h<=50, -49<=k<=41, -41<=l<=41
Reflections collected / unique	198691 / 49119 [R(int) = 0.0492]
Completeness to θ = 25.242	99.7%
Absorption correction	Empirical
Max. and min. transmission	0.745 and 0.533
Refinement method	Full-matrix least-squares on F^2
Data / restraints / parameters	49119 / 0 / 2515
Goodness-of-fit on F^2	1.123
R indices (all data)	R1 = 0.1130, wR2 = 0.2176
Extinction coefficient	n/a
Largest diff. peak and hole	2.80 and -3.74 e.Å^{-3}

Table S7: Crystallographic Data for Compound **3**

Identification code	JWP4251
Empirical formula	C12 H161 K11 Mo30 O146 S30 Te3
Formula weight	7295.30
Temperature	150(2)K
Wavelength	0.71073Å
Crystal system, space group	Monoclinic, C 2/c
Unit cell dimensions	a = 52.465(4)Å α = 90°<br>b = 37.585(3)Å β = 114.426(3)°<br>c = 24.8017(17)Å γ = 90°
Volume	44529(5)$Å^3$
Z, Calculated density	8, 2.176Mg/m^3
Absorption coefficient	2.587 mm^{-1}
F(000)	28048
Crystal size	0.100 x 0.070 x 0.050mm
θ range for data collection	1.646 to 26.000°
Limiting indices	-64<=h<=57, -46<=k<=41, -29<=l<=30
Reflections collected / unique	319733 / 43763 [R(int) = 0.0467]
Completeness to θ = 25.242	100.0%
Absorption correction	Empirical
Max. and min. transmission	0.745 and 0.571
Refinement method	Full-matrix least-squares on F^2
Data / restraints / parameters	43763 / 0 / 2079
Goodness-of-fit on F^2	1.136
R indices (all data)	R1 = 0.0827, wR2 = 0.1789
Extinction coefficient	n/a
Largest diff. peak and hole	1.89 and -1.68 e.$Å^{-3}$

Table S8: Crystallographic data for Compound **4**

Identification code	JWP5101
Empirical formula	C24 H150 K6 Mo24 N2 O119 S24 Te2
Formula weight	5933.25
Temperature	150(2)K
Wavelength	0.71073Å
Crystal system, space group	Tetragonal, I 4 c m
Unit cell dimensions	a = 32.5258(19)Å alpha = 90°<br>b = 32.5258(19)Å beta = 90°<br>c = 29.9125(18)Å gamma = 90.000(10)°.
Volume	31645(4)Å^3
Z, Calculated density	8, 2.491Mg/m^3
Absorption coefficient	2.772mm^{-1}
F(000)	22960
Crystal size	0.100 x 0.050 x 0.050mm
θ range for data collection	0.925 to 25.999°
Limiting indices	-40<=h<=40, -40<=k<=40, -31<=l<=36
Reflections collected / unique	234838 / 15534 [R(int) = 0.0578]
Completeness to θ = 25.242	99.9%
Absorption correction	Empirical
Max. and min. transmission	0.729 and 0.538
Refinement method	Full-matrix least-squares on F^2
Data / restraints / parameters	15534 / 1 / 932
Goodness-of-fit on F^2	1.113
R indices (all data)	R1 = 0.0694, wR2 = 0.1716
Absolute Structure Parameter	0.006(10)
Extinction coefficient	n/a
Largest diff. peak and hole	5.36 and -1.46 e.A^{-3}

Reaction Conditions

Table S9: Selection of reaction conditions that resulted in Compound **1**. The selection included the extremities of yield, manually set pH and crystallisation pH

Squarate content (g)	Tellurite content (g)	Dimer content (mL)	Manually Set pH	Crystallisation pH	Yield (%)
0.099	0.0504	5	3.32	4.92	56.88
0.1002	0.0500	5	4.07	2.86	6.82
0.1003	0.0499	5	1.44	1.1	23.33
0.1003	0.0501	5	5.00	5.52	56.46
0.1020	0.0500	5	5.05	4.4	46.31
0.0995	0.0501	5	1.50	1.46	17.58

Table S10: Selection of reaction conditions that resulted in Compound **2**. The selection included the extremities of yield, manually set pH and crystallisation pH

Squarate content (g)	Tellurite content (g)	Dimer content (mL)	Manually Set pH	Crystallisation pH	Yield (%)
0.1008	0.0507	6.5	5.15	7.17	54.87
0.797	0.497	5	5.32	7.5	3.86
0.1006	0.0497	6.5	5.39	7.87	53.95
0.1004	0.0498	5	4.88	7.01	32.69
0.1004	0.050	6.5	5.36	7.69	41.84
0.1005	0.0506	6.5	5.03	6.07	53.04

Table S11: Selection of reaction conditions that resulted in Compound **3**. The selection included the extremities of yield, manually set pH and crystallisation pH

Squarate content (g)	Tellurite content (g)	Dimer content (mL)	Manually Set pH	Crystallisation pH	Yield (%)
0.1006	0.0501	6.5	5.2	7.6	58.38
0.0192	0.0375	7.5	6.64	8.59	3.52
0.0384	0.075	10	7.00	7.47	6.6
0.0999	0.0501	6.5	5.18	8.01	21.98
0.1000	0.0501	5	6.07	8.38	6.6
0.1004	0.0502	6.5	5.26	7.5	42.82

Table S12: Selection of reaction conditions that resulted in Compound **4**. The selection included the extremities of yield, manually set pH and crystallisation pH

Squarate content (g)	Tellurite content (g)	Dimer content (mL)	Manually Set pH	Crystallisation pH	Yield (%)
0.1000	0.0497	5	3.5	2.98	32.41
0.1005	0.0499	5	4.29	2.52	5.8
0.1020	0.0501	5	4.30	2.96	6.11
0.1000	0.0497	5	3.40	2.72	29.93
0.1010	0.0498	5	4.28	4.14	6.93
0.1003	0.0508	5	3.86	3.75	31.88
